# Perfusion-CT - Can We Predict Acute Pancreatitis Outcome within the First 24 Hours from the Onset of Symptoms?

**DOI:** 10.1371/journal.pone.0146965

**Published:** 2016-01-19

**Authors:** Joanna Pieńkowska, Katarzyna Gwoździewicz, Katarzyna Skrobisz-Balandowska, Iwona Marek, Justyna Kostro, Edyta Szurowska, Michał Studniarek

**Affiliations:** 1 II Department of Radiology–Faculty of Health Sciences, Medical University of Gdansk, Gdansk, Poland; 2 I Department of Radiology–Faculty of Medicine, Medical University of Gdansk, Gdansk, Poland; 3 Department of Gastroenterology and Hepatology, Medical University of Gdansk, Gdansk, Poland; 4 Department of General Endocrine and Transplant Surgery, Medical University of Gdansk, Gdansk, Poland; 5 Department of Diagnostic Imaging, Medical University of Warsaw, Warsaw, Poland; University of Verona, ITALY

## Abstract

**Purpose:**

Severe acute pancreatitis (AP) is still a significant clinical problem which is associated with a highly mortality. The aim of this study was the evaluation of prognostic value of CT regional perfusion measurement performed on the first day of onset of symptoms of AP, in assessing the risk of developing severe form of acute pancreatitis.

**Material and Methods:**

79 patients with clinical symptoms and biochemical criteria indicative of acute pancreatitis (acute upper abdominal pain, elevated levels of serum amylase and lipase) underwent perfusion CT within 24 hours after onset of symptoms. The follow-up examinations were performed after 4–6 days to detect progression of the disease. Perfusion parameters were compared in 41 people who developed severe form of AP (pancreatic and/or peripancreatic tissue necrosis) with parameters in 38 consecutive patients in whom course of AP was mild. Blood flow, blood volume, mean transit time and permeability surface area product were calculated in the three anatomic pancreatic subdivisions (head, body and tail). At the same time the patient's clinical status was assessed by APACHE II score and laboratory parameters such as CRP, serum lipase and amylase, AST, ALT, GGT, ALP and bilirubin were compared.

**Results:**

Statistical differences in the perfusion parameters between the group of patients with mild and severe AP were shown. Blood flow, blood volume and mean transit time were significantly lower and permeability surface area product was significantly higher in patients who develop severe acute pancreatitis and presence of pancreatic and/or peripancreatic necrosis due to pancreatic ischemia. There were no statistically significant differences between the two groups in terms of evaluated on admission severity of pancreatitis assessed using APACHE II score and laboratory tests.

**Conclusions:**

CT perfusion is a very useful indicator for prediction and selection patients in early stages of acute pancreatitis who are at risk of developing pancreatic and/or peripancreatic necrosis already on the first day of the onset of symptoms and can be used for treatment planning and monitoring of therapy of acute pancreatitis. Early suspicion of possible pancreatic necrosis both on the basis of scores based on clinical status and laboratory tests have low predictive value.

## Introduction

Acute pancreatitis is an inflammatory condition that is not limited to pancreas but may also extend to tissues in the vicinity of pancreas. In addition, systemic inflammatory response may also affect other organs [1–6].

The annual incidence of acute pancreatitis ranges from 5 to 80 cases per 100,000 population, with the total mortality of 2–10% [4, 7, 8]. A constant rise in morbidity is likely to be caused by increase in alcohol intake and is estimated as 2.7% a year [9]. The highest increase in prevalence of acute pancreatitis has been reported in young women aged <35 years (7.9% annually) and men aged 35–44 (5.7%) and aged 45–54 (5.3%) [9].

In the majority of patients–ca. in 75% of cases—acute pancreatitis is mild and self-limiting and conservative treatment is sufficient for patients to make a full recovery [3–5, 10, 11]. In such cases, the inflammatory reaction is limited and no concomitant systemic symptoms or multi organ failure occurs. The mortality rate in mild acute pancreatitis is about 0–1%. In 20–25% of patients, the mechanisms responsible for limiting the local inflammatory reaction fail. This leads to necrosis and systemic inflammatory reaction syndrome that can turn into multi organ failure. Such type of disease is classified as severe acute pancreatitis (based on revised Atlanta classification from 2012) and is associated with high mortality ranging from 15 to 25% despite progress in diagnostic tools and treatment [1–3, 5, 12–17]. The extent of necrosis of pancreas and surrounding tissues correlates with patient’s clinical state and is associated with systemic complications.

The diagnosis of acute pancreatitis can be established based on clinical (abdominal pain, nausea and vomiting) and biochemical criteria (elevated pancreatic enzymes–amylase and lipase in plasma at least 3 times exceeding normal variation) as well as imaging.

Contrast-enhanced computed tomography (CT) is regarded as the gold standard imaging modality for diagnosis of acute pancreatitis. A number of prognostic scores based on CT results have been developed to help distinguish cases at risk of severe presentation and increased probability of complications (most popular–Balthazar score) [14, 18–22]. It is vital to diagnose necrosis which occurs in 15–20% of all cases of acute pancreatitis. Necrosis has a prognostic value because in such a group the mortality rate reaches 23% and rises in case of infected necrosis, which is observed, according to authors, in 30–70% of cases. In severe acute pancreatitis, the necrosis of pancreas and surrounding tissues can lead to shock, hypovolemia, acute respiratory distress syndrome (ARDS), disseminated intravascular coagulation (DIC), and renal failure, and may also affect colon and cause fistulas [11, 23–27].

Pancreatic necrosis associated with severe acute pancreatitis usually occurs within 72 hours from the onset of disease. CT scans may be equivocal within 24–48 hours. It is thus recommended to perform CT scanning after 72 hours from the onset of symptoms [28].

Clinical scores used to assess severity of the disease and to establish prognosis are usually insufficient in the first period of pancreatitis. This may lead to delays in the introduction of adequate intensive treatment.

Implementation of hypoperfusion assessing protocol, by attaching CT-perfusion protocol, which makes it possible to demonstrate areas of pancreatic perfusion abnormalities (hypoperfusion), to a standard CT protocol for evaluating pancreatitis, we can predict further course of the disease and help to take appropriate therapeutic decisions.

Perfusion computed tomography (p-CT) is a relatively novel diagnostic tool, which uses hemodynamic data obtained from tissues and organs [7, 29–36]. Due to the fact that pancreatic microcirculation in acute pancreatitis is severely affected, it is believed that, compared with standard CT, this tool should be more sensitive in determining areas in pancreas with blood flow impairment, where pancreatic necrosis may possibly develop. Referring patients with acute pancreatitis for perfusion CT soon after they have been admitted to hospital and diagnosing pancreatic perfusion abnormalities we can have impact on the decisions made with regard to their further therapeutic process.

The aim of the study was to assess prognostic parameters of perfusion CT scan such as blood flow, blood volume, mean transit time and vascular permeability surface, performed on the first day of onset of symptoms in patients with severe acute pancreatitis. In addition, we analyzed evolution of changes in the hypoperfused areas based on the first p-CT and evaluated the impact of perfusion on the development of severe complication of acute pancreatitis–necrosis.

## Material and Methods

Independent Bioethics Commission for Research by Medical University of Gdańsk specifically approved this study—approval number 275/2009. All participants provided written consent for CT examination with intravenous contrast administration which was taken routinely after their admission to the hospital with suspicion of acute pancreatitis. This consent procedure was approved by bioethics commission.

### Patients

Patients with a clinical suspicion of acute pancreatitis who were admitted to emergency department in the years 2010–2013 underwent perfusion CT on the first day on their hospitalization. We enrolled 79 patients aged 25–86 years (mean age 43 years): 32 women and 47 men. The diagnosis of acute pancreatitis was established based on symptoms such as acute abdomen, nausea, vomiting and elevated levels of amylase and lipase in laboratory tests.

### US, clinical and biochemical evaluation

Before admission, all the patients had had abdominal ultrasound exams to exclude cholecystolithiasis and/or ductal gallstones and splenic and/or portal vein thrombosis. The patients’ severity of the disease was assessed using APACHE II score—initially and after 4–6 days. In addition, parameters such as C-reactive protein (CRP), serum lipase and amylase, urine amylase, aspartate transaminase (AST), alanine transaminase (ALT), gamma-glutamyl transpeptidase (GGT), alkaline phosphatase (ALP) and bilirubin were compared. The exclusion criteria were past history of reaction to iodinated contrast agents, pregnancy, and elevated serum creatinine level.

Intensive symptomatic management with intravenous prophylactic antibiotics or basic treatment with a few-day strict dietary restrictions, restoring water-electrolyte balance and pain management were implemented in patients depending on the predicted course of the disease.

The diet was initiated and gradually extended once the pain had eased and the pancreatic parameters normalized, and in the case of severe acute pancreatitis patients were administered enteral feeding through a nasojejunal tube.

### CT examinations

**Fig 1:**
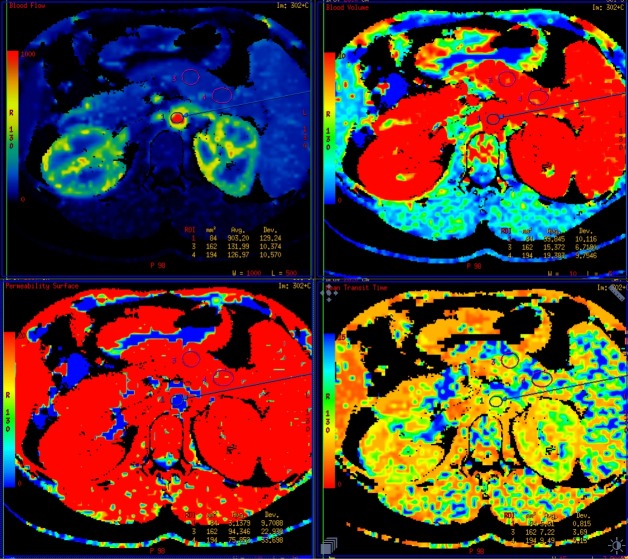
Colour perfusion maps for blood flow (BF), blood volume (BV), permeability surface (PS) and mean transit time (MTT).

CT scans were performed on the first day of acute onset of inflammation (in accordance with the perfusion protocol) and on 4th-6th day of the disease (standard method) with 64-slice CT scanner (GE Light Speed VCT USA). All the patients had a local perfusion assessment involving evaluation of the following parameters: blood flow (BF) (ml/100g/min), blood volume (BV) (ml/100g of tissue), mean transit time (MTT)—which is defined as time needed for the blood to be transferred through vascular bed in seconds—and permeability surface (PS) for the contrast material to move from the intracellular to extracellular space (ml/100g/min) (Fig 1).

Evaluation of pancreatic perfusion was performed in a 4-cm thick section for a period of 40 seconds after a bolus injection of 40 mL low-osmolar contrast media (450 mg iodine/dL) using automated contrast injector (Nemoto Dual Shot Alpha) at a rate of 4 mL/s and a delay of 12 s, obtaining 40 images for every 5-mm thick section. Intravenous administration of contrast agent was heated before injection to 35 degrees Celsius using infusion fluid warming cabinet (EmTherm 1D) and was followed by a saline chase at the same infusion rate (20 mL at the rate of 4 mL/s). The following parameters of p-CT scanning were implemented: tube voltage of 100 kV, tube current of 80 mAs, collimation 64x0.6 mm, rotation time 1s, field of view (FOV) 50 cm, matrix 512x512 mm, 5 mm reconstructed section thickness, DLP (mGy-cm)– 737,31.

Pre-enhancement images presented the range of images before arrival of the bolus of contrast agent (before artery input). Depending on the dynamics of the blood flow in the aorta and condition of the pancreas, the number of non-contrast-enhanced time points ranged from 12 to 24. Between the last pre-enhancement image and the last post enhancement image there was located a 40-second time period during which the evaluation of pancreatic perfusion was performed. The temporal resolution was one time point per second. The latest time point after start of the contrast injection depended on the number of non-contrast–enhanced time points, which is connected with the dynamics of blood flow.

Scans were performed in supine position under free breathing and patients were asked to breathe quietly during the examination to minimize motion artefacts. Also to avoid artefacts, patients were informed of possible flushing sensation during contrast agent injection. Like most researchers we did not use either Buscopan/Glucagon to restrict bowel movement or oral contrast agent.

In our examination the cranio-caudal CT scan coverage was limited to 40 mm. Scan location had to be carefully chosen to cover as much of the pancreas as possible. Therefore after scout image a limited non contrast scan of upper abdomen was performed to localize the pancreas and to determine the scan range of the perfusion CT. In cases where the entire pancreas could not covered, only the most peripheral portion of the tail of pancreas was outside the scan.

In the pancreas, 9 oval or round fields were manually selected to form regions of interest (ROI) for perfusion measurements. Three out of nine measurements were located in head, body and tail of pancreas respectively. Large vessels and principal pancreatic duct in case of its enlargement were omitted. The ROIs size was dependent on the size of pancreas and ranged in head from 50 to 233 mm^2^ (mean value 134 mm^2^), in body from 53 to 251 mm^2^ (mean value 130 mm^2^) and in tail from 47 to 295 mm^2^ (mean value 127 mm^2^). Round ROI with diameter from 70 to 120 mm^2^ (mean value 95 mm^2^) placed manually in abdominal aorta was set as a reference point. Measurements of quantitative parameters of tissue perfusion were performed using standard software CT Perfusion 4 (GE Healthcare USA) on a dedicated workstation (Advantage Windows 4.4, GE Healthcare Technologies). Arterial enhancement curve and perfusion color mapping were generated automatically for each pixel representing the value of the studied parameter using maximum slope method algorithm and modified Johnson-Wilson mathematical model (GE Healthcare, Waukesha, WI) which allows simultaneous determination of blood flow, blood volume, mean transit time and permeability surface area product.

After 4–6 days from the onset of symptoms, a standard CT of pancreas was performed in all patients after intravenous injection of 80 ml of low-osmolar contrast media administered at a rate of 3.5 mL/s, in arterial and pancreatic parenchymal phase covering pancreatic field and in portal venous phase routinely covering abdominal cavity in 5-mm thick sections, with reconstructions interval of 2.5 mm. We used variable mAs (automatic matching mAs depending on the weight of the patient).

Perfusion parameters obtained during the first day of the disease were compared between those patients in whom in control, standard CT scan (after 4–6 days) a pancreatic and/or peripancreatic necrosis occurred and those who did not develop necrosis. Quantitative assessment of perfusion parameters was performed to evaluate the impact of acquired variables on the probability of necrosis development—a severe acute pancreatitis complication, and their variations during the treatment.

### Statistical analysis

All statistical analyses were performed using Statsoft Inc. STATISTICA (data analysis software system) version 10.0. Quantitative variables were described using arithmetic mean, standard deviation, median, minimal and maximal value (range) and 95% confidence interval (CI). Qualitative variables were presented as size and percentage.

Shapiro Wilk test was used to determine whether quantitative variable came from the population with normal distribution. Levene’s test was used to assess the variances homogeneity. T-student test for independent samples was applied to assess significant differences between two groups (or in case of unequal variances–Welch’s test) and Mann Whitney U test was used when t-student test was not applicable and for variables in ordinal scale. Significant differences between more than two groups were assessed using F-test (ANOVA) and Kruskal–Wallis analysis was applied when ANOVA was not applicable. When significant changes between groups were observed, post hoc analyses were implemented (Tukey’s range test for F-test and Dunn’s test for Kruskal-Wallis test).

In case of two related variables, t-Student test was used, whereas Wilcoxon signed-rank test was applied when t-Student test was not applicable and for variables in ordinal scale. Significant differences between more than two related variables were tested with repeated measures analysis of variance or Friedman test (when repeated measures analysis of variance was not applicable or in case of variables measured in ordinal scale).

A chi-square test was used for qualitative variables (with Yates' correction for continuity when one cell of the table had an expected count smaller than 10, Cochran’s Q test, Fisher's exact test).

To determine the strength and direction of the relationship between variables, correlation analysis was performed, which involved calculation of Pearson and/or Spearman’s correlation coefficient. In all calculation, *P* values of less than 0.05 were considered to be statistically significant.

Study protocol was approved by the local bioethics committee. Informed consent was given by all study participants.

The study was financially supported by the National Science Centre, Poland (grant no G-86).

## Results

### Patients

In the control standard CT of 41 out of 79 patients we found progression of pancreatic or peripancreatic tissue necrosis. In the remaining 38 no progress of the disease with necrotic changes was noted. The mean age of the patients without progression was 46.5 years (range: 25–86 years) and in the patients with progression 47.8 years (range 25–77 years). The percentage of women in the group without progression was significantly higher in comparison to the percentage of women in the progression group (63.2% vs 19.5%, respectively; p = 0.0001).

Acute pancreatitis in patients without progression was related to: cholelithiasis (50%, 19 patients), alcohol abuse (28.9%, 11 patients) and side effect of endoscopic retrograde cholangiopancreatography (10.5%, 4 patients). Injury caused acute pancreatitis in one patient, while in 3 patients the etiology remained unknown. In the group with progression, the main cause of acute pancreatitis was alcohol abuse– 65.9% (27 patients). Cholelithiasis was found in 9.8% (4 patients) and in next 5 patients concominant alcohol and cholelithiasis was shown. Hyperlipidemia was the cause of acute pancreatitis in 2 patients. In the remaining 3 cases the etiology was unknown. There was a significantly higher percentage of patients with cholecystolithiasis and cholelithiasis in the group without progression.

### US, clinical and biochemical evaluation

Pancreatitc ultrasonography on admission was normal in 73.7% of patients in the group without progression and in 29.3% in the group with progression. In the rest, pancreas was not visualized due to bowel gas. The percentage of patients with not visualized pancreas was significantly higher in the group with progression (p = 0.0001).

Thrombosis was significantly more often observed in patients with progression–there were 6 cases of splenic vein thrombosis and 2 cases of splenic and portal vein thrombosis (19.5% patients in this group).

In laboratory tests on admission, only C-reactive protein (CRP) level was significantly higher in the group with progression in comparison to the group without progression [113.17mg/l (range 2.4–398.3) vs.19.83 mg/l (range 0.6–227.2) respectively; p = 0.0001). In laboratory tests conducted along with control CT, CRP level was significantly higher in the group with progression in comparison to the group without progression [133.9 mg/l (range 27.2–294.5) vs. 73.82 mg/l (0.6–251.7); p = 0.0005). Despite lack of significant differences in the levels of lipase and amylase in serum and amylase in urine between the two groups on admission, there was a significant fall in the levels of all biochemical parameters in both groups in the control check-up.

In control CT, there were no statistically significant differences between the two groups in terms of evaluated on admission severity of pancreatitis assessed using APACHE II score.

### Perfusion-CT

In our work, in the pancreas, 9 oval or round fields were selected to form regions of interest (ROI) for perfusion measurements. Three of the nine measurements were taken in the head, three in the body and three in the tail of pancreas. The ROIs size was determined manually and depended on pancreas size: in head it ranged from 50 to 233 mm^2^, in body from 53 to 251 mm^2^ and in tail from 47 to 295 mm^2^. Perfusion assessments depending on ROIs size did not significantly differ and thus the values were averaged. Mean ROI size in head of pancreas was 134 mm^2^, in body 130 mm^2^ and in tail 127 mm^2^.

We noted significantly higher levels of blood flow (BF), blood volume (BV) and mean transit time (MTT) in each part of pancreas (head, body and tail) in the whole group without progression and, simultaneously, significantly higher values of permeability surface (PS) in each part of pancreas in the whole progression group. For this reason the three consecutive measurements for each part of the pancreas (head, body and tail) were averaged.

The mean values of BF for head, body and tail of pancreas were significantly higher (p = 0.0001) in the group without progression in comparison to the progression group and were as follows: head of pancreas: mean 126.4 (±34.9) ml/100g/min (range 49.5–200.8; median 122.6), body of pancreas: mean 125.0 (±30.6) ml/100g/min (range 38.7–178.0; median 129.7), tail of pancreas: mean 131.7 (± 34.4) ml/100g/min (range 61.5–191.4; median 128.5).

**Fig 2:**
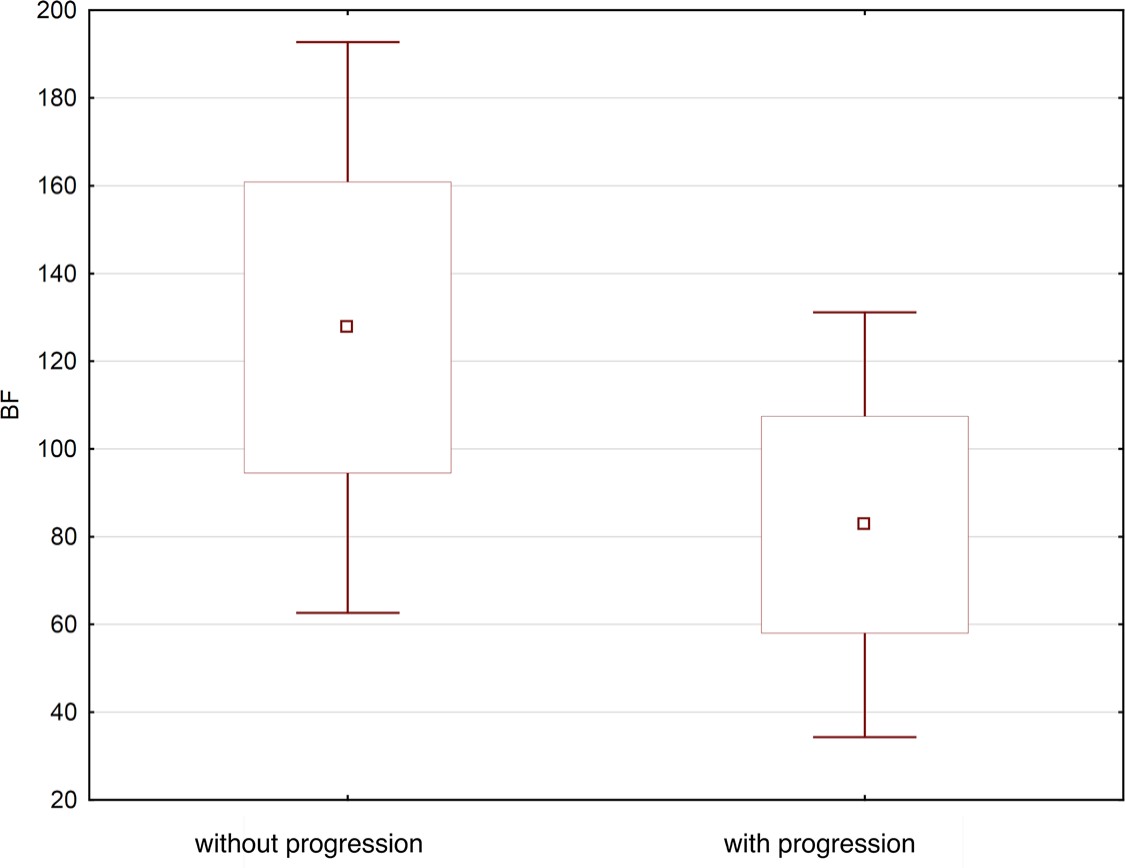
Average BF value in patients with progression and without progression acute pancreatitis illustrated as a box-whiskers-plot graph.

In patients with progression, the BF parameter values were as follows: head of pancreas: mean 79.1 (±21.1) ml/100g/min (range 36.4–131.9; median 76.1), body of pancreas mean 81.9 (± 21.1) ml/100g/min (range 50.3–132.4; median 80.1), tail of pancreas: mean 87.1 (± 30.6) ml/100g/min (range 36.5–162.7; median 80.0) (Fig 2).

Mean BV values for head, body and tail of pancreas were significantly (p = 0.0001) higher in patients without progression in comparison to patients with progression and were as follows: head of pancreas: mean 19.7 (±6.0) ml/100g (range 10.0–31.4; median 18.6), body of pancreas: mean 19.3 (±6.1) ml/100g (range 6.9–31.3; median 17.8), tail of pancreas mean 20.1 (± 6.1) ml/100g (range 8.5–33.7; median 19.0).

**Fig 3:**
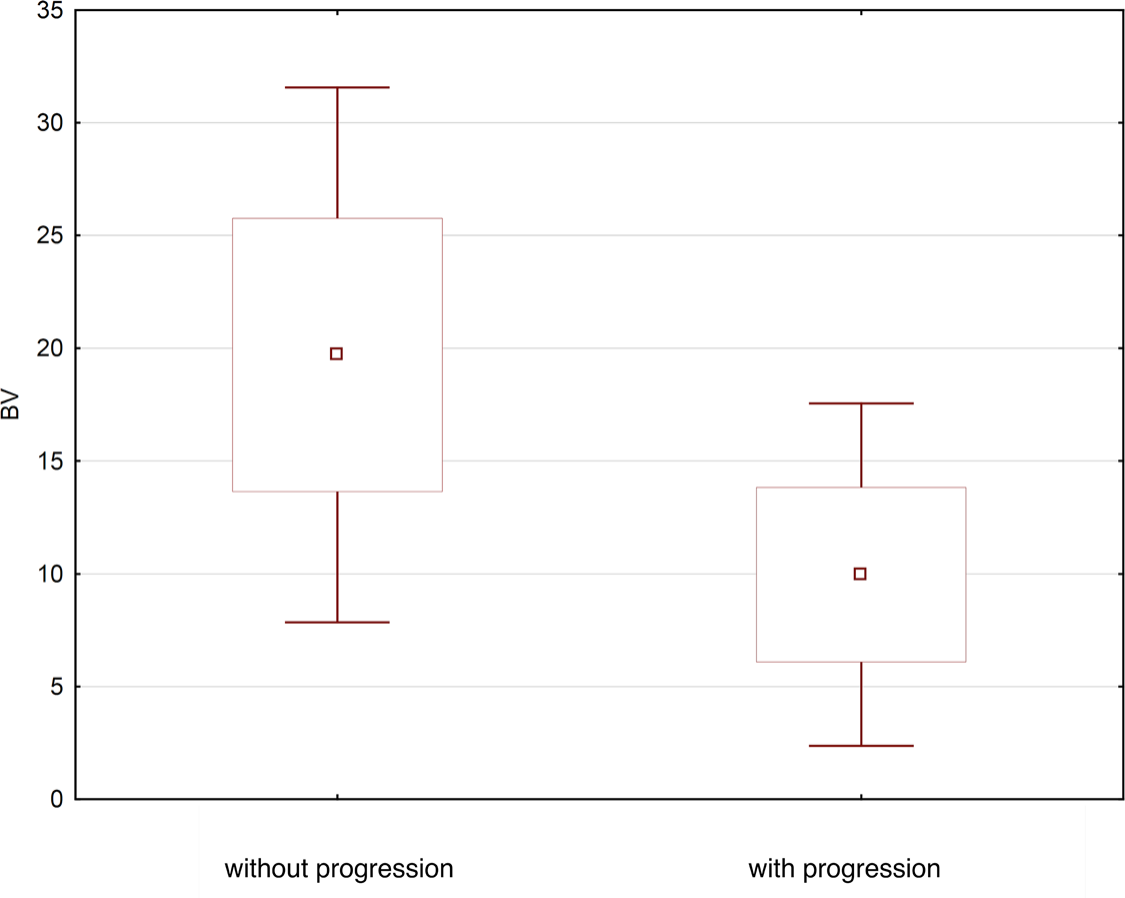
Average BV value in patients with progression and without progression acute pancreatitis illustrated as a box-whiskers-plot graph.

In patients with progression, the BV values were, respectively: head of pancreas range 10.1 (±3.5) ml/100g (range 5.8–19.2; median 9.2), body of pancreas mean 9.5 (± 3.6) ml/100g (range 4.8–20.8; median 9.0), tail of pancreas mean 10.3 (± 4.5) ml/100g (range 4.6–25.3; median 8.4) (Fig 3).

The mean MTT values for head, body and tail of pancreas were significantly (p = 0.0001) higher in patients without progression in comparison to patients with progression and were as follows: head of pancreas: mean 10.9 (±2.3) s (range 6.0–16.6; median 10.5), body of pancreas mean 10.8 (±2.6) s (range 6.0–17.1; median 10.1), tail of pancreas mean 10.5 (± 2.6) s (range 5.4–15.3; median 10.0).

**Fig 4:**
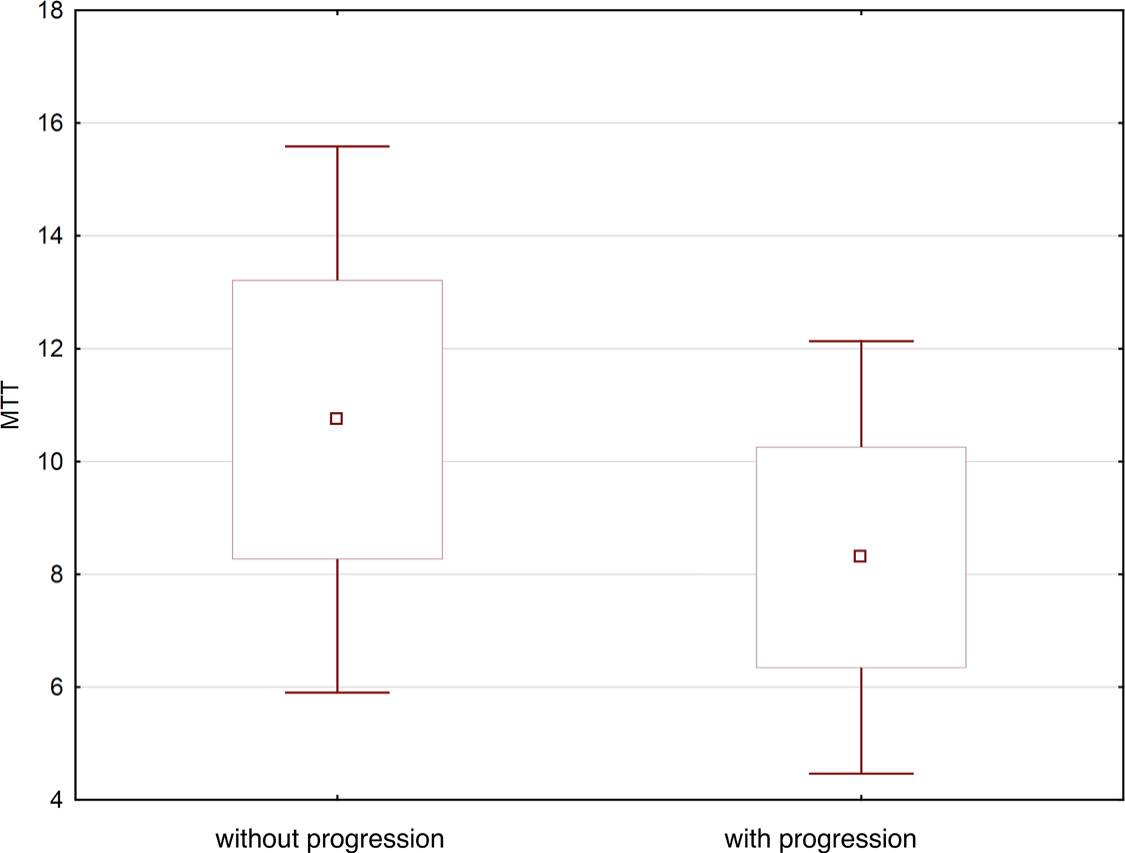
Average MTT value in patients with progression and without progression acute pancreatitis illustrated as a box-whiskers-plot graph.

In patients with progression, the MTT values were as follows: head of pancreas range 8.7 (±2.2) s (range 5.2–14.1; median 8.8), body of pancreas mean 7.9 (± 1.9) s (range 4.6–12.8; median 7.8), tail of pancreas mean 8.3 (± 1.7) s (range 4.9–12.1; median 8.3) (Fig 4).

The mean PS values for head, body and tail of pancreas were significantly (p = 0.0001) lower in patients without progression in comparison to patients with progression and were as follows: head of pancreas: mean 28.3 (±11.2) ml/100g/min (range 8.8–53.3; median 27.1), body of pancreas mean 27.1 (±13.4) ml/100g/min (range 10.7–62.6; median 25.0), tail of pancreas mean 25.9 (± 14.0) ml/100g/min (range 8.7–69.4; median 21.4).

**Fig 5:**
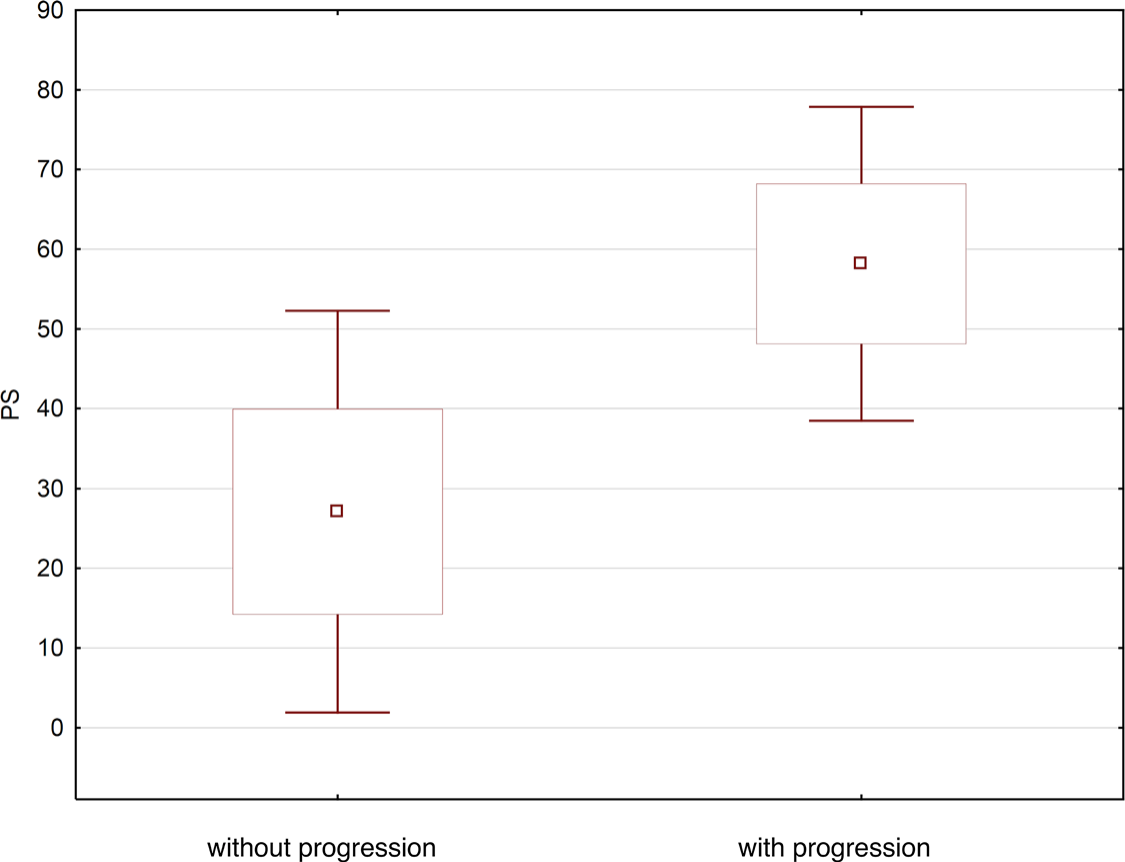
Average PS value in patients with progression and without progression acute pancreatitis illustrated as a box-whiskers-plot graph.

In patients with progression, the PS values were as follows: head of pancreas mean 58.0 (±10.8) ml/100g/min (range 40.1–86.5; median 57.3), body of pancreas mean 58.7 (± 10.2) ml/100g/min (range 42.2–83.3; median 58.9), tail of pancreas mean 57.9 (± 9.3) ml/100g/min (range 43.1–83.7; median 55.4) (Fig 5).

In order to predict most accurately pancreatic necrosis, cut-off points were set for each perfusion parameter (BF, BV, MTT, PS) based on ROC curve (Table 1).

**Table 1:** Analysis of sensitivity, specificity and ROC for averaged BF, BV, MTT and PS for head, body and tail of pancreas with regard to progression or lack of progression

	BF	BV	MTT	PS
Head				
AUC	0.874	0.927	0.767	0.969
95%CI	[0.790;0.957]	[0.874;0.981]	[0.663;0.871]	[0.937;1.00]
Suggested cut-off point	96.904	12.788	9.721	40.111
Sensitivity	87.8%	82.9%	70.7%	100.0%
Specificity	81.6%	92.1%	73.7%	84.2%
Body				
AUC	0.879	0.920	0.819	0.954
95%CI	[0.795;0.962]	[0.854;0.985]	[0.728;0.910]	[0.903;1.0]
Suggested cut-off point	93.343	12,719	8,672	42,222
Sensitivity	75.6%	90,2%	73,2%	100,0%
Specificity	92.1%	89,5%	81,6%	92,1%
Tail				
AUC	0.825	0.916	0.760	0.963
95%CI	[0.733;0.918]	[0.850;0.982]	[0.653;0,867]	[0.914;1.0]
Suggested cut-off point	95.120	14.515	9.315	43.094
Sensitivity	70.7%	90.2%	75.6%	100.0%
Specificity	89.5%	92.1%	73.7%	89.5%

Based on ROC curve, the cut-off points for BF were as follows: head of pancreas 96.904 mL/100mL/min, body of pancreas 93.343 mL/100mL/min, tail of pancreas 95.120 mL/100mL/min. With such levels of BF, necrosis development in each part of pancreas (head, body and tail) may be predicted with sensitivity of 87.8%, 75.6% and 70.7% respectively and specificity of 81.6%, 92.1% and 89.5% respectively.

In case of BV, the cut-off points that may indicate possible pancreatic necrosis development were as follows: head of pancreas 12.788 mL/100mL, body of pancreas 12.719 mL/100mL, tail of pancreas 14.515 mL/100mL. With such levels of BV, necrosis development in each part of pancreas (head, body and tail) may be predicted with sensitivity of 82.9%, 90.2% and 90.2% respectively and specificity of 92.1%, 89.5% and 92.1% respectively.

The cut-off points for MTT that may indicate possible pancreatic necrosis development were as follows: head of pancreas 9.721 s, body of pancreas 8.672 s mL/100mL, tail of pancreas 9.315 mL/100mL. With such levels of MTT, necrosis development in each part of pancreas (head, body and tail) may be predicted with sensitivity of 70.7%, 73.2% and 75.6% respectively and specificity of 73.7%, 81.6% and 73.7% respectively.

Based on ROC curve, the cut-off points for PS were as follows: head of pancreas 40.111 mL/100mL/min, body of pancreas 42.222 mL/100mL/min, tail of pancreas 43.094 mL/100mL/min. With such levels of PS, necrosis development in each part of pancreas (head, body and tail) may be predicted with sensitivity of 100.0%, 100.0% and 100.0% respectively and specificity of 84.2%, 92.1% and 89.5%.respectively.

**Fig 6:**
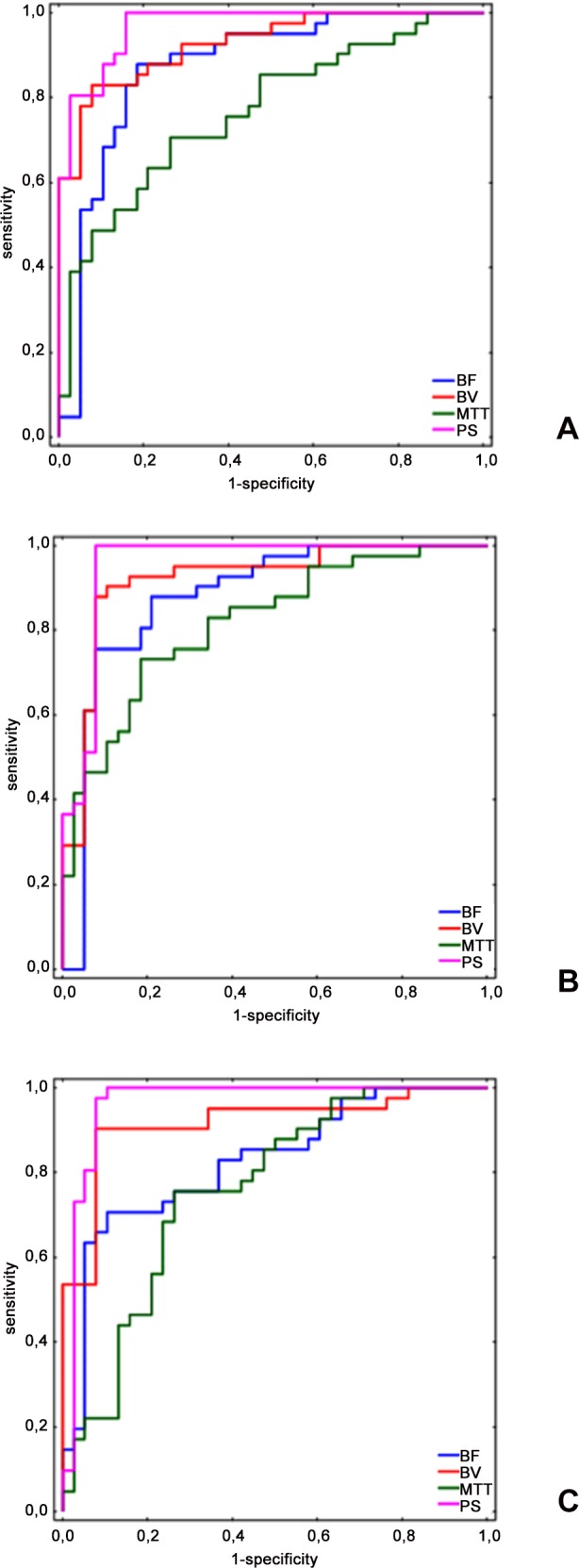
ROC curves for different parts of pancreas: (A) Head of pancreas. (B) Body of pancreas. (C) Tail of pancreas.

The area under ROC curve (AUC), which determines the ability of a test to distinguish between correct and incorrect measurements, is greatest for PS and BV—for head of pancreas 0.969 and 0.927 (excellent) respectively, for body of pancreas 0.954 and 0.920 (excellent) and tail of pancreas 0.963 and 0.916 (excellent) respectively (Fig 6A–6C).

## Discussion

Despite progress in diagnosis and treatment, the mortality rate in severe acute pancreatitis (SAP) still remains high, reaching 15–25% [7, 37–39]. The extent of pancreatic and peripancreatic necrosis is a vital and the most threatening prognostic factor that affects clinical state of patients and makes it possible to predict systemic complications. Early in the course of acute pancreatitis, dynamic development of inflammation with diminished pancreatic and peripancreatic tissues perfusion is observed. There may occur a complete regression of such alterations but they can also progress to irreversible necrosis. Due to high risk of multi organ complications and high mortality rate in patients with SAP, there have been attempts to distinguish patients who are prone to pancreatic necrosis, based on perfusion CT (p-CT) images, in order to intensify their treatment.

The analyzed group consisted of 79 consecutive patients who had a diagnosis of acute pancreatitis based on clinical and laboratory findings. This group was fairly large when compared to other studies. Tsuji [37] evaluated p-CT performed in 30 patients with SAP while Yadav [38] evaluated 53 patients, including 32 with SAP and 21 with mild acute pancreatitis (MAP). In Delerue study [40], only 3 patients out of the 54 who had pancreatic perfusion assessment suffered from acute pancreatitis.

Unlike in majority of available studies concerning this topic, in our study we performed p-CT in all patients on the first day after the onset of acute pancreatitis symptoms. It is believed that evolution of ischemia and necrosis development takes a few days to be noticed in imaging [2, 4, 7, 38, 41]. For this reason, conventional CT with multiple phases of pancreas is recommended after 72 hours from the onset of acute pancreatitis symptoms. Early conventional CT may undervalue the final necrotic area or may even be normal within first 48 hours. In vast majority of studies evaluating perfusion disturbances in inflammation of pancreas, p-CT scans were performed up to 72 hours from the symptoms onset [37, 38, 42, 43]. In one study, the perfusion-CT scans were acquired within the first 24 hours from hospital admission, but it was not specified what time elapsed from the onset of symptoms. In addition, the assessment of severity of pancreatitis using Balthazar score (grade A to E) might indicate longer duration of the disease in some of the analyzed patients [31]. In some publications there was no accurate data concerning time elapsed between the onset of symptoms and p-CT examination.

The aim of our study was to assess the role of perfusion CT in predicting the likelihood of pancreatic necrosis already on the first day after the onset of symptoms. This is the time in which other methods, both imaging and scores based on clinical course and laboratory tests, offer low prognostic value.

In the presented study, every patient had had ultrasonography before admission. In addition, acute pancreatitis was assessed initially and after 4–6 days using APACHE II score. Moreover, parameters such as CRP, pancreatic lipase and amylase in serum, amylase in urine, AST, ALT, GTP, ALP and bilirubin were compared.

Pancreas was not shown in ultrasonography in as many as 70.7% of our patients in the progression group and 26.3% in the group without progression. It is commonly known that the limitation of this method is caused by paralytic ileus that accompanies acute pancreatitis in the first 48 hours of the disease. The role of ultrasonography was thus to exclude cases of cholecystolithiasis and/or bile duct lithiasis and splenic and/or portal vein thrombosis.

In the laboratory tests performed on admission, only mean CRP level (protein synthesized by the liver in many pathological conditions) was significantly higher in the group with progression in comparison to the group without progression (113.17 mg/l vs. 19.83 mg/l respectively, p = 0.0001). Despite statistically significant differences in CRP level already on the first day of the disease, the parameter does not reach its peak in such an early period and cannot be used as a prognostic factor within the first 24 hours. In acute pancreatitis, only CRP level above 210 mg/L that is observed after 48 hours following the onset of the disease is regarded as a prognostic factor of SAP. In recent years, there have been observations that CRP level >150 mg/L within 48 hours of symptoms onset correlates with the expected severity of the acute pancreatitis [4].

Along with inflammatory markers, other laboratory and clinical parameters, such as pancreatic enzymes activity in serum and urine, respiratory system and renal function, body temperature, neurological status, complete blood count and others used in scores designated to define the severity of clinical state, are not helpful in the early phase in distinguishing patients who will develop SAP. A three-fold increase of the upper limit of normal value of pancreatic amylase or lipase in serum combined with clinical findings is regarded as diagnostic for acute pancreatitis. Serum amylase and lipase levels, however, do not correlate with severity of the disease. The elevated enzymes are also not specific for acute pancreatitis and may be observed in myocardial infarction, acute cholecystitis, intestinal obstruction, intestinal ischemia, perforated gastric or duodenal ulcer or renal failure [4, 5, 44]. Moreover, it is possible that in patients with necrosis affecting the entire pancreas, no significantly elevated levels of pancreatic enzymes are observed in the advanced stage. Such presentation of the disease may usually be seen in patients with alcohol abuse.

Our study also did not reveal any significant differences in pancreatic amylase and lipase in serum and amylase in urine levels between patients with and without progression in control CT. Moreover, no statistically significant differences between the two groups of patients were observed in the severity of acute pancreatitis assessed on the first day of the disease using APACHE II score. Multiparametric scores of clinical state have been used for many years to predict further course of the disease and distinguish patients at risk of pancreatic necrosis who would benefit from early intensive treatment. These include: Glasgow score, Ranson’s criteria and APACHE II score [23, 45]. Three or more points in Ranson’s criteria and eight or more points in APACHE II score may suggest SAP [1–3, 5, 8]. APACHE score enables selection of patients within the first 48–96 hours of the disease. None of those scores, however, is sufficient to differentiate patients at the time of hospital admission. It has been proved that the earlier the identification of patients at risk of SAP the better the treatment results [4, 5, 7, 46].

Based on the presented results, it seems that risk stratification in acute pancreatitis and assessment of unfavorable course of the disease will be possible using p-CT already on the first day when the symptoms occur.

Inflammatory process in pancreas is usually diffused and affects the entire gland [5]. For this reason, we performed three measurements in each pancreatic area (head, body and tail); the obtained results were averaged. We found significant differences in all perfusion parameters (BF, BV, MTT and PS) between patients without acute pancreatitis progression and those with progression of the disease. The discrepancies with regard to the differences in the values of BF and BV parameters observed in our study when compared with other available data may be due to the implementation of different algorithms by different authors to calculate/analyze perfusion parameters [29].

Different mathematical kinetic models are used in the assessment of pancreatic perfusion. This is due to the fact that different CT manufacturers installed different software solutions. Commonly used perfusion algorithms are single or dual compartment models: maximum slope method, deconvolution method and Patlac method. This is the reason why comparison of the measured values of perfusion parameters and standardization of CT perfusion is hindered. The perfusion model used in our software is based on a distributed version of a compartmental model, which was developed with capillary permeability (modified Johnson-Wilson model). This model allows simultaneous determination of blood flow, blood volume, mean transit time and permeability-surface area product.

The most common algorithms used in other study were: maximum slope method [31, 34, 40], deconvolution method [37], single-compartment method [47], and the Patlak method [35, 38, 48]. Another explanation of the differences in BF and BV could be the fact that we performed the measurements earlier in comparison to other studies, i.e. already on the first day of the disease. In our study we did not relate BF and BV in pancreas to the same parameters in liver as a control organ, as was done in some studies [37, 38]. We believe that it is wrong to assume that circulation in liver was not disturbed as a result of pancreatic inflammation.

The other two parameters, MTT and PS, have not been evaluated in other studies concerning acute pancreatitis [31, 37] or there have been no differences between patients with regard to those parameters [38].

Similarly to other studies, we found significantly higher mean values of BF for head, body and tail of pancreas in patients without progression when compared to those with progression (p = 0.0001). In patients with mild, edematous acute pancreatitis mean BF in head, body and tail of pancreas were: 126.4 ± 34.9 mL/100g/min, 125.0 ± 30.6 mL/100g/min and 131.7 ± 34.4 ml/100g/min, respectively. In patients with progression, the observed mean BF values for head, body and tail of pancreas were: 79.1 ± 21.1 mL/100g/min, 81.9 ± 21.1 ml/100g/min and 87.1 ± 30.6 mL/100g/min, respectively.

Also mean BV values for head, body and tail of pancreas were significantly higher (p = 0.0001) in patients without progression in comparison with patients with progression. The mean BV for head, body and tail of pancreas in patients without progression was: 19.7 ± 6 mL/100g, 19.3 ± 6.1 mL/100g and 20.1 ± 6.1 mL/100g, respectively. In patients with progression, in whom pancreatic and/or peripancreatic necrosis occurred, mean BV values for head, body and tail of pancreas were: 10.1 ± 3.5 mL/100g, 9.5 ± 3.6 mL/100g and 10.3 ± 4.5 mL/100g, respectively.

All authors agree that both in acute and chronic pancreatitis BF and BV measurements are significantly lower than in normal pancreatic gland, in which mean BF is 70–90 mL/min/100g according to Patlak analysis, 80–130 mL/min/100g using maximum slope method, and 120-140ml/min/100g using deconvolution technique [30, 34–36, 40, 47–49].

Decrease in BF and BV may be explained by cellular edema, thrombosis of the pancreatic microcirculation with resultant diminished microcirculatory function, and by vasospasm [13, 30, 37, 38, 50, 51].

The BV value observed in our study was similar to Bize’s study [31], in which the same perfusion analysis algorithm was applied. In that study, the mean BV in patients with MAP was 23.7 mL/100mL, while in patients with SAP BV level of 10.4 mL/100mL was noted. Interestingly, BF in patients without progression measured on the first day of the disease was high, similar to the one observed in normal pancreas. In patients with necrosis, BF value was low already on the first day of the inflammatory process. Both values are higher than those measured by other authors in the later stage of the disease [38, 37].

It is not completely clear for us why we did not observe differences in perfusion parameters value between individual parts of the pancreas (head, body and tail), in the progression group, although at a later stage of the disease necrosis affected various parts of the pancreas and /or peripancreatic tissue.

In our study, perfusion abnormalities concerned the whole organ.

Perhaps, this is due to the early performed perfusion-CT, up to 24 hours from the onset of the symptoms when necrotic lesions are not yet clearly defined.

It is possible that in such an early period of acute pancreatitis it is difficult to predict how local circulation in the pancreas will behave under reduced blood flow and exactly which areas of pancreas will undergo necrosis.

However, this is a very interesting issue that requires further study.

Mean transit time (MTT) is a parameter that describes mean time needed for contrast media to pass through tissue. In the presented study, the mean MTT for head, body and tail was significantly higher (p = 0.0001) in patients without progression when compared to those with progression. Mean MTT for head, body and tail of pancreas in patients without progression was: 10.9 ± 2.3 s, 10.8 ± 2.6s and 10.5 ± 2.6s, respectively. In patients with progression of the disease mean MTT for head, body and tail of pancreas was: 8.7 ± 2.2s, 7.9 ± 1.9s and 8.3 ± 1.7s, respectively. This parameter has not been evaluated in the majority of studies concerning acute pancreatitis. In one study, there were no significant differences between patients with and without necrosis, but no accurate data concerning the level of the measured parameter was presented [38]. We cannot thus compare our results with any other study. We noted significantly lower levels of mean transit time in the whole group with progression of acute pancreatitis, which means that MTT was longer in patients with mild acute pancreatitis (MAP) compared to patients with severe acute pancreatitis (SAP).

It seems that significantly longer mean presence time of contrast media in pancreatic parenchyma in MAP may be caused by lower permeability of intrapancreatic vessels for contrast media passing from intra to extracellular space, which is associated with lower degree of pancreatic gland dysfunction, in which perfusion alterations eventually disappear in the later stage of the disease. Because the value of MTT was obtained by dividing BV by BF, the progressive cellular edema of the pancreas in the inflammatory process may also explain the relatively greater reduction in the value of BV and associated reduction in the value of MTT.

The last of the evaluated parameters, permeability surface area product, was also not assessed in other studies concerning acute pancreatitis [31, 37], or there were no significant differences between specific groups of patients [38]. In many studies, however, permeability has been assessed in healthy pancreatic tissue in which mean PS was 28 ± 14 ml/min/100g using maximum slope method [30, 34–36, 40, 47–49]. The mean values of PS observed in our study for head, body and tail of pancreas were significantly lower (p = 0.0001) in patients without progression in control assessment in comparison with patient with progression. The mean PS for head, body and tail of pancreas in patients without progression of acute pancreatitis was: 28.3 ± 11.2 mL/100g/min, 27.1 ± 13.4 mL/100g/min and 25.9 ± 14.0 mL/100g/min, respectively. In the group of patients with progression, in which pancreatic and/or peripancreatic necrosis occurred, PS level was significantly higher and in head, body and tail of pancreas reached: 58.0 ± 10.8 mL/100g/min, 58.7 ± 10.2 mL/100g/min and 57.9 ± 9.3 mL/100g/min, respectively. In patients without progression, PS value measured on the first day of the disease was low, similar to the value observed in normal pancreatic parenchyma, calculated using the same algorithm for perfusion analysis. Patients who developed necrosis in the later stage of the disease, had significantly higher level of PS already on the first day of acute pancreatitis. It is possible that lack of significant differences in PS level in Yadav’s study [38] may be the consequence of late p-CT scanning, which was performed up to 72 hours after the symptoms onset. Cut-off points based on ROC curves that aimed at better prediction of pancreatic necrosis already within the first 24 hours of the disease indicate that PS has the best predictive value and the highest sensitivity in that period. Cut-off points value based on ROC curves for PS in head, body and tail of pancreas were 40.111 mL/100mL/min, 42.222 mL/100mL/min, 43.094 mL/100mL/min, respectively. With such PS values, necrosis in all regions of pancreas can be predicted with 100% sensitivity and specificity of 84.2%, 92.1% and 89.5% for head, body and tail, respectively.

The results obtained in our study have to be confirmed in other studies performed in the first 24 hours from the onset of clinical symptoms of acute pancreatitis, also using the other perfusion analysis algorithms.

The drawback of the study was, like in every CT, the use of radiation. According to European studies, mean effective dose for CT perfusion is 3.54 mSv [7]. However, research is still conducted on the use of low dose CT scans to reduce radiation dose acquired by patient while maintaining satisfactory pancreatic images [22, 48, 49, 52]. Significant progress in this field is expected to be achieved in the coming years. Use of MR perfusion would allow us to avoid the negative effect of ionizing radiation. However, MR imaging is time-consuming and considering the poor condition of patients with SAP it may be difficult to perform due to impaired cooperation with the patient and the medical equipment maintaining the patient's vital functions. Therefore the use of MRI is limited in severely ill patients and is considered to be the first-choice imaging procedure in pregnant women and in patient with allergy to iodinated contrast or with renal failure.

The use of iodinated contrast media is also disadvantageous for the patients. It has been proved in animal models that those media may exacerbate acute pancreatitis, increase number of complications and prolong recovery period by deteriorating pancreatic microcirculation. This has not, however, been confirmed in humans [5, 37, 53]. P-CT is more favorable when compared with standard multiphase pancreatic CT due to the fact that it requires less contrast media (only 40 ml).

## Conclusion

In conclusion, taking into consideration the results of our study, it seems that p-CT is a method that may reveal pancreatic perfusion abnormalities. Consequently, it helps to distinguish patients who are likely to develop pancreatic and/or peripancreatic necrosis already on the first day of the onset of symptoms. P-CT should be potentially useful for early administration of adequate treatment that could prevent the development of irreversible pancreatic necrosis. Early suspicion of possible pancreatic necrosis, both on the basis of standard CT, scores based on clinical status and laboratory tests have low predictive value.
